# Fluoxetine Repurposing Mitigates Alzheimer’s Disease Pathology via the GSK3β–CREB–ADAM10 Axis

**DOI:** 10.3390/ijms27062676

**Published:** 2026-03-14

**Authors:** Soo-Ho Lee, Yeonghoon Son, Hyosun Jang, Hyun-Yong Kim, Kwang Seok Kim, Hyun-Shik Lee, Hae-June Lee

**Affiliations:** 1 Division of Radiation Biomedical Research, Korea Institute of Radiological & Medical Sciences, Seoul 01812, Republic of Korea; skarn88@naver.com (S.-H.L.);; 2 KNU G-LAMP Project Group, KNU Institute of Basic Sciences, School of Life Sciences, College of Natural Sciences, Kyungpook National University, Daegu 41566, Republic of Korea; 3 BK21 FOUR KNU Creative BioResearch Group, School of Life Sciences, College of Natural Sciences, Kyungpook National University, Daegu 41566, Republic of Korea; 4 New Drug Development Center, Osong Medical Innovation Foundation, Cheongju 28160, Republic of Korea; 5 Veterinary Medical Research Institute, College of Veterinary Medicine, Jeju National University, Jeju 63243, Republic of Korea

**Keywords:** fluoxetine, Alzheimer’s disease, cognitive impairment, p-CREB, α-secretase

## Abstract

Alzheimer’s disease (AD) is the most prevalent neurodegenerative disorder in the aging population. Drug repurposing provides a cost-effective strategy to identify novel therapeutics that may mitigate age-associated pathologies. Here, we report the therapeutic potential of fluoxetine, a selective serotonin reuptake inhibitor commonly used as an antidepressant, in alleviating cognitive impairment and AD-like pathology in 5xFAD mice, a transgenic model of familial AD. Chronic fluoxetine administration significantly ameliorated anxiety-like behavior and cognitive deficits in 5xFAD mice, as assessed by open field, Y-maze, and novel object recognition tests. Fluoxetine treatment was associated with reduced amyloid plaque deposition in the hippocampus and cortex, attenuation of microglial activation, and decreased expression of inflammatory cytokines. At the molecular level, fluoxetine increased phosphorylation of GSK3β at Ser9, which was associated with enhanced CREB phosphorylation and upregulation of the α-secretase ADAM10. These effects were further examined in SH-SY5Y neuronal cells, where CREB phosphorylation and ADAM10 expression were significantly modulated by GSK3β inhibition, whereas CaMKII inhibition had no detectable effect under our experimental conditions. Our findings suggest that fluoxetine modulates amyloid-associated signaling pathways in the 5xFAD model, in part through regulation of the GSK3β-CREB signaling framework. These results provide mechanistic insight into how fluoxetine may influence APP processing in an amyloid-driven pathological context, although further studies are required to clarify its translational implications in human AD.

## 1. Introduction

Alzheimer’s disease (AD) is the most common neurodegenerative disorder in individuals over the age of 65 years. With the global increase in life expectancy, the number of individuals affected by dementia is projected to rise from the 57 million in 2019 to 153 million by 2050 [1]. AD is a progressive and multifactorial disease involving several environmental and genetic factors, including apolipoproteins [2] and triggering receptor expressed on myeloid cells 2 (TREM2) [3]. Pathologically, AD is characterized by beta-amyloid (Aβ) deposition and the formation of tau-neurofibrillary tangles in the brain, leading to synaptic dysfunction and cognitive decline that significantly impacts quality of life [4]. AD is broadly classified into sporadic and familial forms based on family history. While some patients report a positive family history (often referred to as familial clustering), strictly defined autosomal dominant familial AD caused by mutations in APP, PSEN1, or PSEN2 is rare and accounts for less than 2% of total AD cases [5,6,7]. Although uncommon, autosomal dominant forms of AD have provided important mechanistic insight into amyloid-driven pathogenesis and have served as a foundation for experimental modeling. The vast majority of AD cases are sporadic and likely arise from complex interactions among aging, genetic susceptibility factors, and environmental influences; however, the underlying pathophysiology remains incompletely understood. Among various risk factors, aging remains most prominent [8].

There is an urgent need for effective therapeutic strategies to prevent or delay AD progression. However, the development of new drug is a complex, costly, and time-consuming process, and numerous attempts over the past decades have failed to yield effective treatments [9]. An alternative strategy, drug repurposing, offers a promising approach because their pharmacokinetics and safety profiles of approved drugs are well established.

Fluoxetine, marketed as Prozac, is a selective serotonin reuptake inhibitor (SSRI) approved by the U.S. Food and Drug Administration (FDA). It increases synaptic serotonin levels by inhibiting serotonin reuptake transporters and presynaptic autoreceptors [10]. Fluoxetine has also been reported to exert anti-inflammatory effects, particularly under conditions of chronic stress or inflammation [11], suggesting potential relevance in neuroinflammation disorder such as AD. Chronic fluoxetine treatment has been shown to enhance brain-derived neurotrophic factor (BDNF) expression in limbic and dopaminergic brain regions, supporting synaptic plasticity [12,13]. Moreover, fluoxetine has been reported to exert neuroprotective, antioxidant, and anti-inflammatory effects in both normal and AD animal model [14]. However, some preclinical and clinical studies have demonstrated limited or inconsistent therapeutic benefit [15,16]. Recent clinical findings indicate that SSRI treatment may be associated with reduced plasma p-tau181 levels and altered serotonergic network activity [17]. Preclinical studies have also suggested that fluoxetine may influence AD-related pathology through modulation of antioxidant and inflammasome pathways [18]. Given the complexity and heterogeneity of these findings, the precise molecular mechanisms by which fluoxetine may influence AD-related pathology remain incompletely understood. In the present study, we investigated the therapeutic effects and underlying mechanism of fluoxetine using a familial AD mouse model and SH-SY5Y neuronal cells.

## 2. Results

### 2.1. Fluoxetine Improves Anxiety-like Behavior and Memory Deficits in 5xFAD Mice

**Figure 1:**
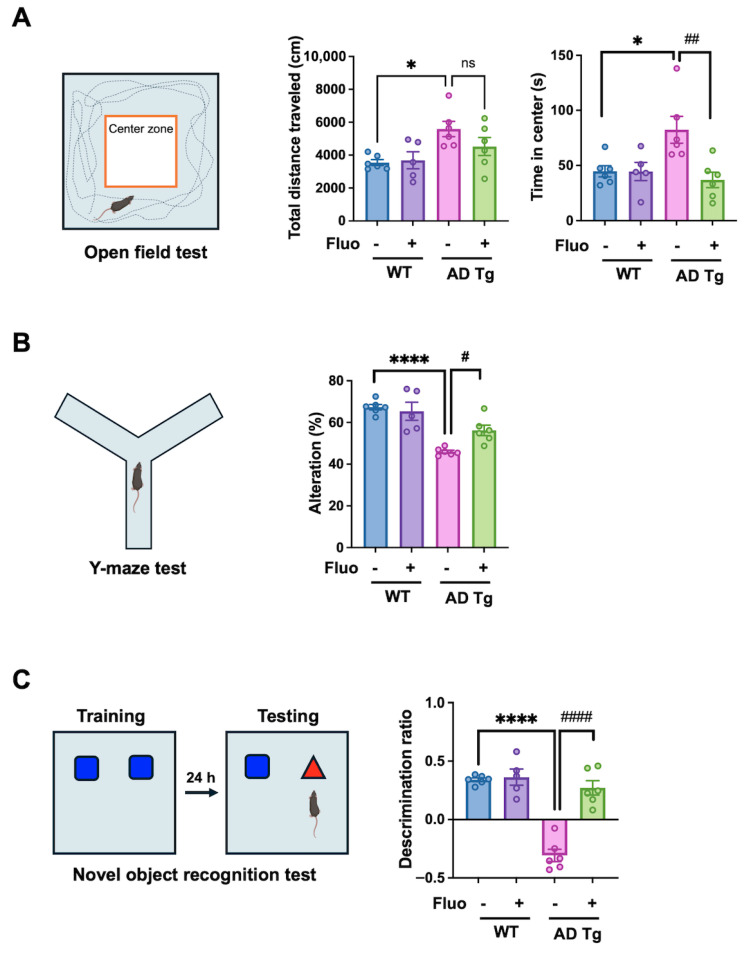
Effect of fluoxetine on behavioral performance in wild-type and 5xFAD transgenic mice (AD Tg). (**A**) Open-field test assessing general locomotor activity and anxiety-like behavior based on total distance traveled and time spent in the center zone. (**B**) Y-maze test evaluating spontaneous alteration behavior as an index of spatial working. (**C**) Novel object recognition (NOR) test assessing recognition memory. Mice were exposed to two identical objects (blue square) during the training phase, and then to one familiar and one novel object (red triangle) during the testing phase conducted 24 h later. Data are represented as mean ± SEM. Sample sizes were as follows: WT + vehicle (*n* = 6), WT + fluoxetine (*n* = 5), AD Tg + vehicle (*n* = 6), and AD Tg + fluoxetine (*n* = 6). Statistical significance was determined using one-way ANOVA followed by Tukey’s post hoc test. * *p* < 0.05, **** *p* < 0.0001 vs. WT + vehicle; ^#^ *p* < 0.05, ^##^ *p* < 0.01, ^####^ *p* < 0.0001 vs. AD Tg + vehicle; ns, not significant.

To assess the effects of fluoxetine on behavioral phenotypes, wild-type (WT) and 5xFAD transgenic (AD Tg) mice were subjected to behavioral tests. In the open-field test, the total distance traveled was significantly increased in AD Tg + vehicle (Veh) mice compared to WT + Veh controls (*p* = 0.0234), whereas fluoxetine administration did not alter locomotor activity in either WT or AD Tg mice. AD Tg mice spent more time in the center zone than WT controls (*p* = 0.0299), suggesting altered anxiety-related behavior. This change was significantly normalized following fluoxetine treatment in AD Tg mice (*p* = 0.0077; Figure 1A).

In the Y-maze test, AD Tg + Veh mice showed a significant reduction in spontaneous alteration compared with WT controls (*p* < 0.0001), indicating impaired spatial working memory. Fluoxetine treatment significantly rescued this behavior in AD Tg mice (*p* = 0.0288; Figure 1B). In the novel object recognition test, AD Tg + Veh showed a decreased discrimination ratio compared with WT + Veh controls (*p* < 0.0001), suggesting impaired recognition memory. This deficit was significantly improved in the AD Tg + Fluo group (*p* < 0.0001; Figure 1C). These findings indicate that chronic fluoxetine treatment improves anxiety-related and cognitive behavioral performance in 5xFAD mice.

### 2.2. Fluoxetine Reduces Amyloid Pathology in the Brain of 5xFAD Mice

**Figure 2:**
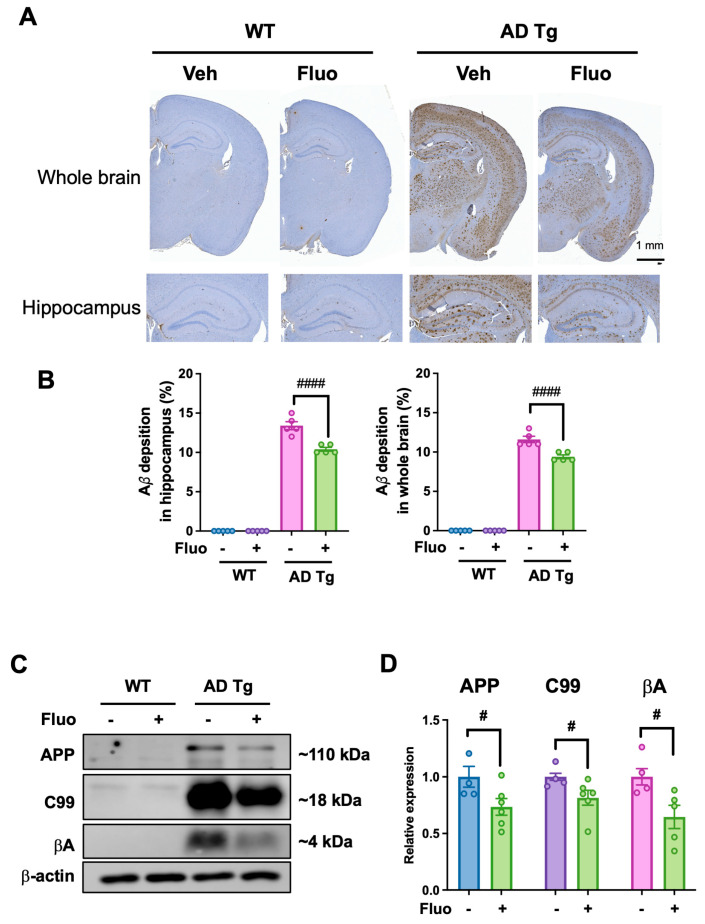

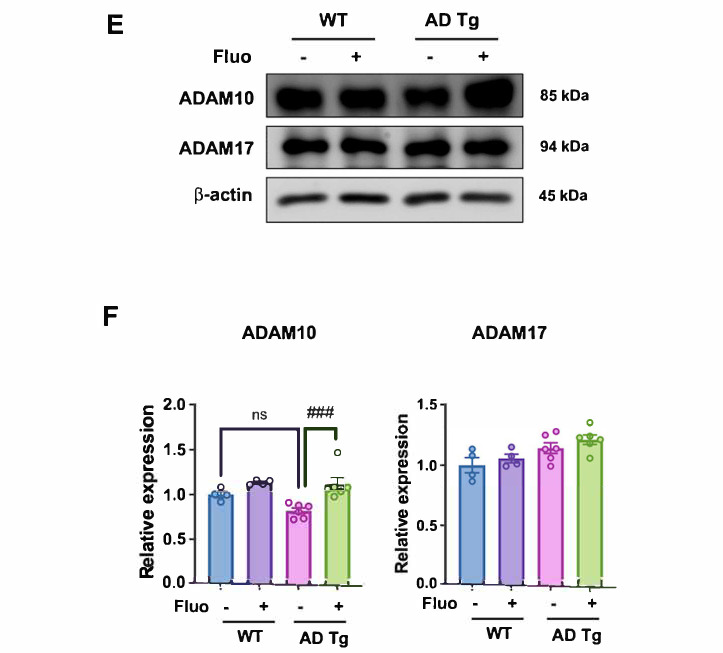
Fluoxetine is associated with reduced amyloid pathology in 5xFAD transgenic mice. (**A**) Representative images of Aβ immunostaining (6E10 antibody) in the hippocampus and whole brain hemisphere of wild-type and 5xFAD mice treated with fluoxetine (Fluo) or vehicle (Veh). (**B**) Quantification of Aβ-positive area as a percentage of total analyzed region in the hippocampus and whole brain hemisphere. Data are presented as mean ± SEM. Sample sizes for immunohistochemistry were as follows: WT + vehicle (*n* = 5), WT + fluoxetine (*n* = 5), AD Tg + vehicle (*n* = 5), and AD Tg + fluoxetine (*n* = 5). Individual data points represent biological replicates. (**C**) Representative Western blot analysis using 6E10 antibody to detect full-length APP (~110 kDa), C99 (~18 kDa), and Aβ (~4 kDa). (**D**) Quantitation of APP, C99 and Aβ protein levels normalized to β-actin. WT groups showed minimal background-level 6E10 immunoreactivity; therefore, statistical analysis was restricted to comparisons between AD Tg + vehicle and AD Tg + fluoxetine groups using an unpaired two-tailed Student’s *t*-test for (**B**,**D**). ^#^ *p* < 0.05, ^####^ *p* < 0.0001 vs. AD Tg vehicle. (**E**) Representative Western blot analysis of ADAM10 and ADAM17 protein expression in hippocampal lysates. (**F**) Quantification of ADAM10 and ADAM17 levels normalized to β-actin. Data are presented as mean ± SEM. Sample sizes for Western blot analysis were as follows: WT + vehicle (*n* = 4), WT + fluoxetine (*n* = 4), AD Tg + vehicle (*n* = 5), and AD Tg + fluoxetine (*n* = 5). Individual data points represent biological replicates. Statistical analysis was performed using one-way ANOVA followed by Tukey’s post hoc test. ^###^ *p* < 0.001, vs. AD Tg + vehicle; ns, not significant.

Amyloid plaque deposition was evaluated using immunohistochemistry and Western blot analysis. As expected, substantial Aβ plaque accumulation was observed in the hippocampus and cortex of AD Tg + Veh mice. Fluoxetine treatment significantly reduced the Aβ deposition in both the hippocampus and hemispheric brain sections compared to AD Tg + Veh mice (Figure 2A,B). Western blotting with the 6E10 antibody further confirmed decreased levels of APP (*p* = 0.0295), C99 (*p* = 0.0255), and βA (*p* = 0.0221) in the fluoxetine-treated AD Tg group (Figure 2C,D). To examine whether fluoxetine modulates APP processing enzymes, protein levels of ADAM10 and ADAM17 were determined. Fluoxetine treatment significantly increased ADAM10 expression in the hippocampus (*p* = 0.0009), while ADAM17 levels remained unchanged (Figure 2E,F). These results suggest that fluoxetine may be associated with reduced Aβ burden, potentially through enhanced non-amyloidogenic processing linked to ADAM10 upregulation.

### 2.3. Fluoxetine Suppresses Neuroinflammation in 5xFAD Mice

**Figure 3:**
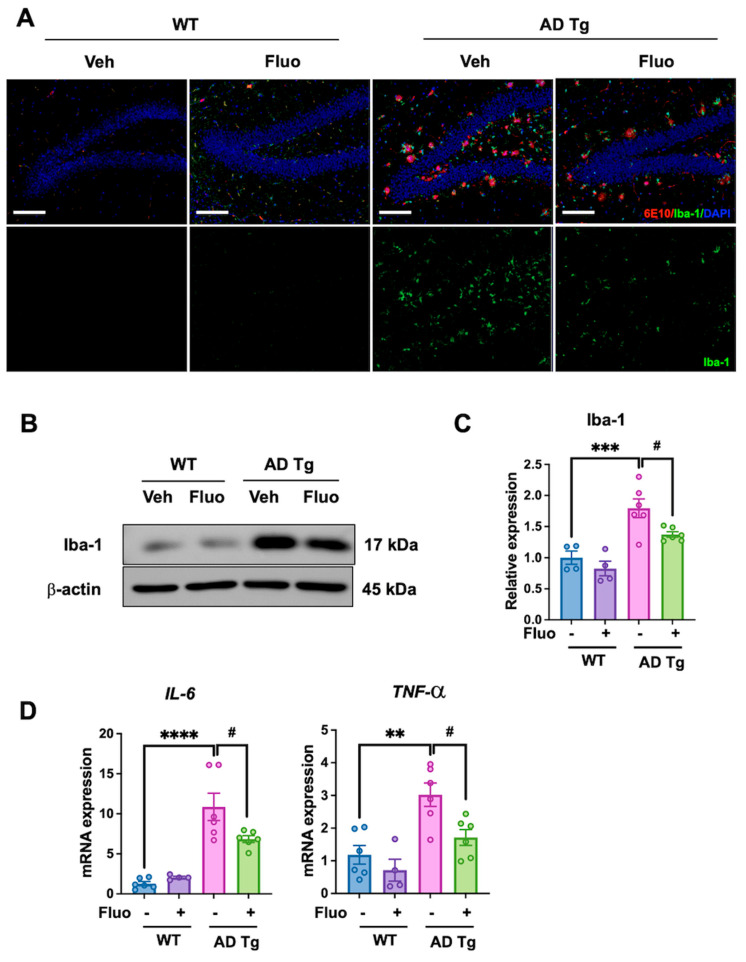
Fluoxetine attenuates neuroinflammatory markers in 5xFAD mice. (**A**) Representative immunofluorescence staining image showing Aβ for (6E10, red) and microglia (Iba-1, green) staining in the hippocampus. Nuclei were counterstained with DAPI (blue). Scale bar = 200 μm. (**B**) Representative Western blot analysis of Iba-1 expression in hippocampal lysates. (**C**) Quantification of Iba-1 protein levels normalized to β-actin. Data are presented as mean ± SEM. Sample sizes for Western blot analysis were as follows: WT + vehicle *(n* = 4), WT + fluoxetine (*n* = 4), AD Tg + vehicle (*n* = 6), and AD Tg + fluoxetine (*n* = 6). (**D**) mRNA expression of proinflammatory cytokines *IL-6* and *TNF-α* in cortex tissue analyzed by quantitative RT-PCR. Data are presented as mean ± SEM. Sample sizes for qPCR were as follows: WT + vehicle (*n* = 6), WT + fluoxetine (*n* = 4), AD Tg + vehicle (*n* = 6), and AD Tg + fluoxetine (*n* = 6). Statistical analysis was performed using one-way ANOVA by Tukey’s post hoc test. ** *p* < 0.01, *** *p* < 0.001, **** *p* < 0.0001 vs. WT + vehicle; ^#^ *p* < 0.05 vs. AD Tg + vehicle.

To investigate the anti-inflammatory effect of fluoxetine, Iba-1 expression was analyzed as a marker of microglial activation. Immunofluorescent staining of hippocampal sections with 6E10 (red) and Iba-1 (green) and DAPI (blue) revealed a marked increase in Iba-1 positive microglia in AD Tg + Veh mice compared to WT groups. Treatment with fluoxetine reduced Iba-1 immunoreactivity in AD Tg mice, indicating attenuation of microglial activation (Figure 3A). Western blot analysis of hippocampus lysates revealed increased Iba-1 expression in AD Tg + Veh mice compared with WT controls (*p* = 0.0002), which was significantly reduced following fluoxetine treatment (*p* = 0.0202; Figure 3B,C). Consistent with this result, RT-qPCR analysis of cortex tissue showed decreased mRNA expression of proinflammatory cytokine *IL-6* (*p* = 0.0415) and *TNF-α* (*p* = 0.0382) in the fluoxetine-treated AD Tg group (Figure 3D). These results indicate that fluoxetine attenuates neuroinflammation in 5xFAD mice. The reduction in Iba-1 and pro-inflammatory cytokines (IL-6, TNF-α) is consistent with attenuation of beta-amyloid induced neuroinflammatory responses.

### 2.4. Fluoxetine Enhances Phosphorylation of Synaptic Signaling Molecules

**Figure 4:**
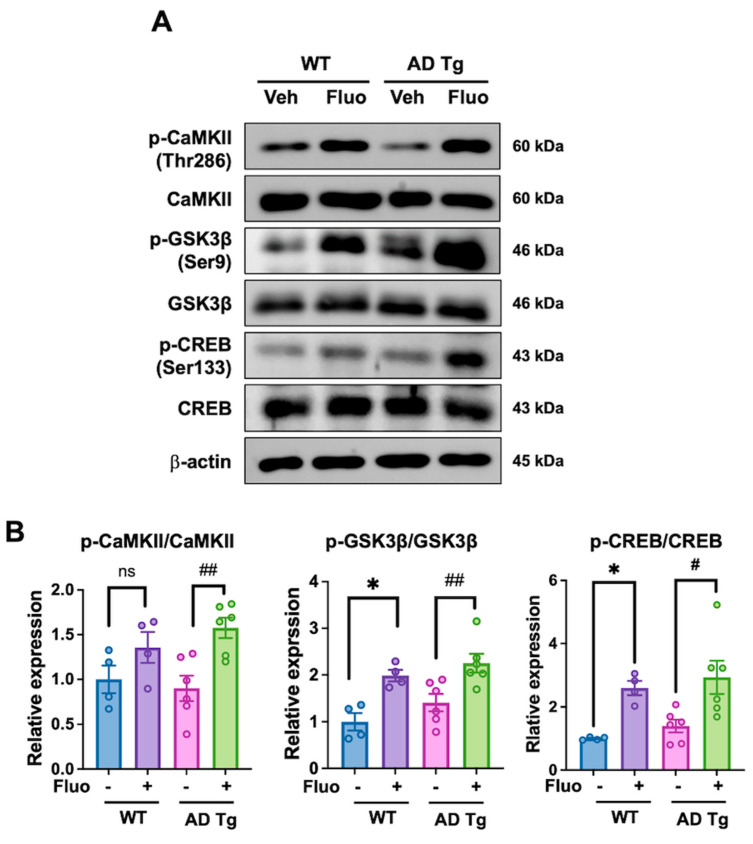

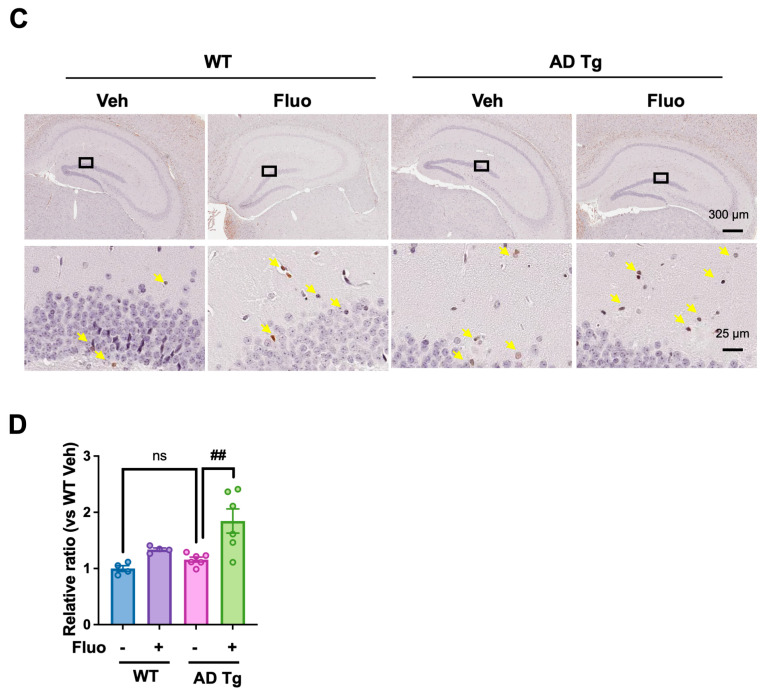
Fluoxetine is associated with enhanced phosphorylation of synaptic signaling molecules in the hippocampus. (**A**) Representative Western blot analysis of phosphorylated and total forms of CaMKII (Thr286), GSK3β (Ser9), and CREB (Ser133) in hippocampal lysates. (**B**) Quantification of phosphorylated proteins normalized to their corresponding total protein levels. (**C**) Representative immunohistochemistry images of p-CREB-positive cells the hippocampal region. Black squares represent the areas selected for enlarged image. Yellow arrows indicate immune-positive nuclei. Scale bar: 300 μm (upper panels), 25 μm (lower panels). (**D**) Quantification of p-CREB-positive cells per defined hippocampal field. Data are presented as mean ± SEM. Sample sizes were as follows: WT + vehicle (*n* = 4), WT + fluoxetine (*n* = 4), AD Tg + vehicle (*n* = 6), and AD Tg + fluoxetine (*n* = 6). Statistical analysis was performed using one-way ANOVA followed by Tukey’s post hoc test (**B**,**D**). * *p* < 0.05 vs. WT + vehicle; ^#^ *p* < 0.05, ^##^ *p* < 0.01 vs. AD Tg + vehicle; ns, not significant.

Since fluoxetine administration improved memory impairment and significantly reduced beta-amyloid deposition in 5xFAD mice, we next determined the activation of signaling pathways associated with synaptic plasticity and memory. Western blot analysis of hippocampus lysates showed increased phosphorylation of CaMKII (Thr286), GSK3β (Ser9), and CREB (Ser133) in both WT and AD Tg mice following fluoxetine treatment (Figure 4A,B). Immunohistochemistry of hippocampus confirmed increased p-CREB in AD Tg + Fluo mice (*p* = 0.0042; Figure 4C,D). These findings are consistent with involvement of the GSK3β–CREB signaling pathway in fluoxetine-associated molecular changes.

### 2.5. Fluoxetine Modulates GSK3β–CREB–ADAM10 Signaling in SH-SY5Y Cells

**Figure 5:**
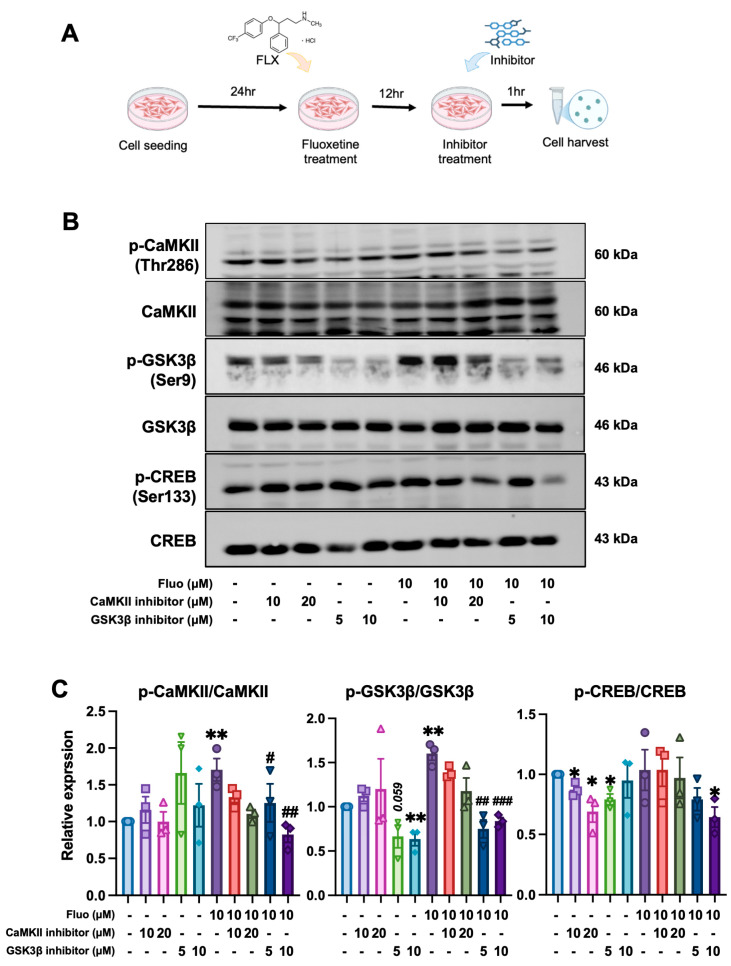

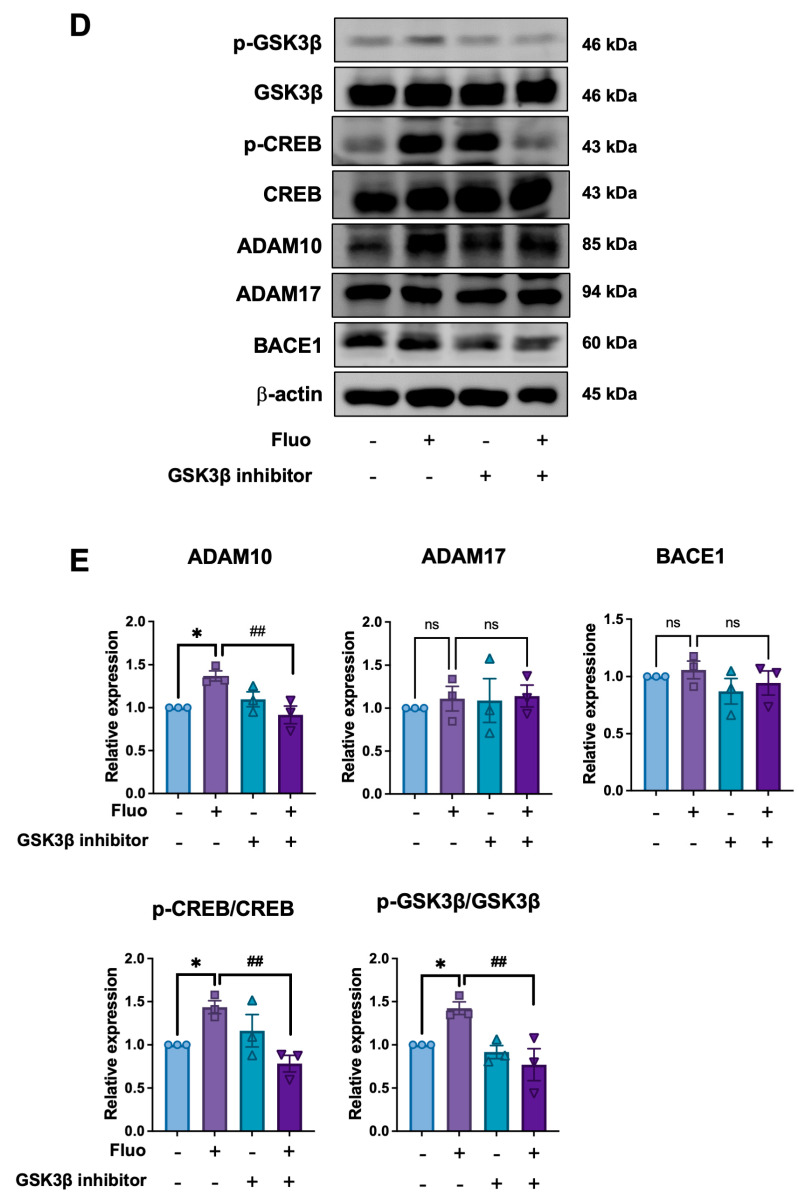
Fluoxetine modulates CaMKII-GSK3β-CREB signaling and ADAM10 expression in vitro. (**A**) Schematic diagram of the experimental design. SH-SY5Y cells were seeded and pretreated with fluoxetine (10 μM) for 12 h, followed by treatment with the CaMKII inhibitor 1-NA-PP1 (10 or 20 μM) or the GSK3β inhibitor IX (5 or 10 μM) for 1 h prior to cell harvest. (**B**) Representative Western blot analysis for phosphorylated and total forms of CaMKII (Thr286), GSK3β (Ser9), and CREB (Ser133). (**C**) Quantification of relative phosphorylation levels normalized to the corresponding total protein. (**D**) Representative Western blot analysis of signaling and APP-processing-related proteins following fluoxetine and/or GSK3β inhibitor treatment. (**E**) Quantification of protein expression levels normalized to β-actin (for protein) or to the corresponding total protein (for phosphorylated forms), as indicated. Data are presented as mean ± SEM (*n* = 3 independent experiments). Individual data points represent independent biological replicates. Statistical analysis was performed using one-way ANOVA followed by Tukey’s post hoc test. * *p* < 0.05, ** *p* < 0.01 vs. control; ^#^ *p* < 0.05, ^##^ *p* < 0.01, ^###^ *p* < 0.001 vs. fluoxetine alone; ns, not significant. Created in BioRender. Son, Y. (2026) https://BioRender.com/s91n646 (accessed on 31 January 2026).

To further examine the molecular mechanism by which fluoxetine regulates the p-CREB activation, SH-SY5Y cells were treated with fluoxetine (10 μM), a GSK3β inhibitor (GSK3β inhibitor IX; 5 or 10 μM), and CaMKII inhibitor (1-NA-PP1; 10 or 20 μM). Fluoxetine treatment significantly increased phosphorylation of GSK3β (*p* = 0.0014) and CREB (*p* = 0.0097), whereas p-CaMKII was not significantly altered (*p* = 0.8405; Figure 5A–C). Co-treatment with fluoxetine and GSK3β inhibitor reversed the fluoxetine-induced upregulation of p-CREB (*p* = 0.0089), while CaMKII inhibition had no effect. These results suggest fluoxetine-induced p-CREB phosphorylation is associated with modulation of GSK3β signaling under our experimental condition. Furthermore, protein analysis showed that fluoxetine increased ADAM10 expression (*p* = 0.0147), while co-treatment with fluoxetine and GSK3β inhibitor attenuated ADAM10 upregulation by fluoxetine (*p* = 0.0048; Figure 5D,E). Expression of ADAM17 and BACE1 remained unchanged. These finding support fluoxetine is associated with increased CREB phosphorylation and ADAM10 expression, potentially involving GSK3β-dependent signaling.

## 3. Discussion

In this study, we explored the therapeutic potential of fluoxetine, a widely used antidepressant, in the context of Alzheimer’s disease (AD). Our findings indicate that chronic fluoxetine treatment is associated with improved behavioral performance and reduced AD-like pathology in 5xFAD mice. While our data support the involvement of the GSK3β–CREB–ADAM10 axis, fluoxetine is known to modulate multiple converging signaling cascades. Therefore, the observed molecular and pathological changes likely reflect coordinated pathway integration rather than a strictly linear cascade. In particular, modulation of the GSK3β–CREB–ADAM10 signaling axis appears to be associated with changes in amyloid burden and behavioral abnormalities.

Fluoxetine significantly alleviated anxiety-like behavior and spatial memory impairment in 5xFAD mice. Results from Y-maze and novel object recognition test suggest that fluoxetine improves both hippocampus-dependent working memory and recognition memory. This observation has clinical relevance given high comorbidity between depressive symptoms and cognitive impairment in patients with AD [19,20]. Chronic fluoxetine administration has been reported to modulate both hippocampal and non-hippocampal memory systems, although its effects may vary depending on the memory phases. For instance, fluoxetine impairs remote memory in some experimental paradigms but does not affect short-term learning, with some deficits reversing after drug withdrawal [21]. Enantiomer-specific effects have also been reported; (R)-fluoxetine enhances cognitive flexibility, spatial learning, and hippocampal neurogenesis to a greater extent than (S)-fluoxetine, suggesting that the cognitive effects of fluoxetine involve both structural and functional neural adaptations [22].

Moreover, fluoxetine has been shown to restore neuroplasticity in the adult brain region beyond the hippocampus, including the visual cortex, likely through proteomic remodeling intracellular signaling and synaptic architecture [23]. These findings support the view that fluoxetine promotes cognitive recovery not merely through antidepressant effects but also by reactivating dormant plasticity mechanisms in the aging or diseased brain. Additionally, fluoxetine has been associated with improvement in social and emotional behavior, particularly under stress-related conditions such as social isolation. Chronic fluoxetine treatment has been shown to reverse deficits in social memory and reduce depressive-like behaviors, potentially via enhanced neurogenesis in the olfactory bulb [24]. Collectively, these findings highlight fluoxetine’s broader neurobiological function, including the promotion of neurogenesis, synaptic plasticity, and cognitive resilience, which are highly relevant to neurodegenerative and mood disorders.

Consistent with these behavioral findings, fluoxetine reduced Aβ plaque deposition in the hippocampus and cortex. This effect was accompanied by increased expression of the α-secretase ADAM10 and decreased levels of the β-amyloid precursor fragment APP and C99. These findings are consistent with enhanced non-amyloidogenic APP processing. However, ADAM10 primarily functions as a constitutive α-secretase, and its impact on overall amyloid burden depends on the dynamic balance between α- and β-secretase activity. Therefore, the reduction in Aβ deposition observed in this study should be interpreted within the broader context of APP processing regulation.

At the molecular level, fluoxetine increased the GSK3β phosphorylation at Ser9, thereby reducing its kinase activity [25] and potentially facilitating CREB phosphorylation and nuclear activity [26]. CREB and CaMKII are key regulators of synaptic plasticity and cognitive function [27], while GSK3β plays a critical role in beta-amyloid production [28]. The observed upregulation of ADAM10 following fluoxetine treatment suggest a mechanistic link between GSK3β inhibition and non-amyloidogenic APP cleavage. This pathway supported by in vitro experiments in SH-SY5Y cells, where GSK3β inhibition reversed CREB and ADAM10 upregulation by fluoxetine, whereas CaMKII inhibition had no effects. These findings suggest that GSK3β contributes substantially to the observed signaling changes under our experimental conditions. However, given that CaMKII activation was assessed primarily using pharmacological inhibition in vitro, additional genetic approaches would be required to definitively exclude its contribution.

**Figure 6:**
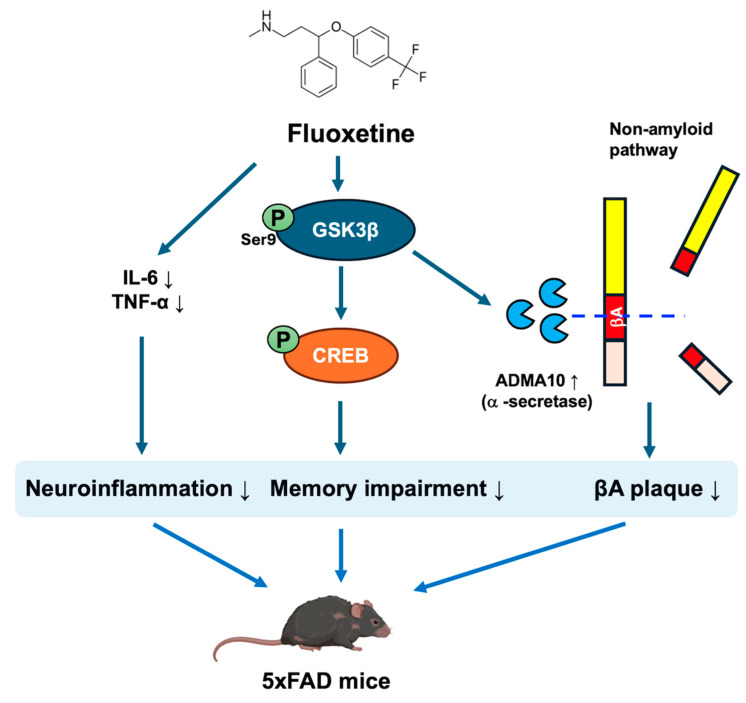
Proposed model of the fluoxetine-mediated signaling pathway in AD. Chronic fluoxetine administration is associated with enhanced non-amyloidogenic processing of amyloid precursor protein (APP) through a GSK3β–CREB–ADAM10 signaling axis. Fluoxetine increases GSK3β phosphorylation at Ser9, which is associated with CREB activation and upregulation of the α-secretase ADAM10. This schematic summarizes the signaling interactions proposed based on the present findings. The model illustrates a potential relationship among GSK3β inhibition, CREB activation, ADAM10 upregulation, and reduced amyloid deposition; however, causal relationships among these events remain to be further established. Fluoxetine treatment was associated with reduced microglial activation and decreased pro-inflammatory cytokines. While these inflammatory changes may be influenced by altered amyloid burden, fluoxetine is also known to exert serotonergic and anti-inflammatory effects through mechanisms independent of APP processing. Therefore, the anti-inflammatory effects observed here may reflect both amyloid-dependent mechanisms and parallel signaling pathways. Created in BioRender. Son, Y. (2026) https://BioRender.com/s91n646 (accessed on 31 January 2026).

Our findings align with previous studies showing that fluoxetine increased Ser9 phosphorylation inactivates GSK3β [29], and phosphorylation of CREB involved in upregulation ADAM10 [30]. This cascade plays a key role in synaptic plasticity and memory-related processes and has been implicated in the regulation of amyloid processing. Fluoxetine-induced inactivation of GSK3β has been linked to increased nuclear β-catenin levels and hippocampal neurogenesis [29], as well as improved cognitive function and oligodendrogenesis in AD mouse models [31]. Furthermore, activation of canonical Wnt/β-catenin signaling by fluoxetine has been reported to reduce Aβ pathology via GSK3β inhibition [32]. These glial and neuronal effects may collectively contribute to cognitive improvement and modulation of amyloid pathology. A schematic of summary of the proposed pathway is presented in Figure 6.

Based on the findings, we propose a working model in which fluoxetine inhibits GSK3β via Ser9 phosphorylation, leading to increased CREB activation and subsequent ADAM10 expression. While reduced amyloid deposition may contribute to behavioral improvements, fluoxetine is also known to influence synaptic plasticity and neurotransmission independently of amyloid pathology. Therefore, the behavioral effects observed in this study may reflect multifactorial mechanisms rather than a single causal pathway.

While ADAM10 can be regulated through multiple signaling pathways, including Wnt/β-catenin and PP2A, clinical trials of SSRIs in established AD have generally reported limited cognitive benefit in patients with established AD [33]. Nevertheless, emerging evidence suggests that fluoxetine may modulate disease-related biomarkers, including plasma p-tau and serotonergic network activity. These findings raise the possibility that the therapeutic impact of fluoxetine may depend on intervention timing and pathological stage rather than symptomatic endpoints alone. Thus, elucidating its molecular mechanisms remains important for defining appropriate translational contexts.

Although our data highlight the involvement of the GSK3β–CREB axis in the 5xFAD model, previous studies have implicated Akt, PP2A, Wnt/β-catenin, ERK, and BDNF signaling in fluoxetine-mediated effects. The relative contribution of these pathways may vary depending on disease stage and pathological context. Accordingly, the GSK3β–CREB–ADAM10 pathway should be considered one component within a broader signaling network. For instance, whereas fluoxetine has been shown to upregulate ADAM10 via the Wnt/β-catenin pathway in 3xTg-AD mice [34], our findings in the 5xFAD model suggest that the GSK3β–CREB axis may represent a regulatory route in models characterized by rapid and aggressive amyloidosis. Recognizing these converging pathways underscores the importance of considering pathological heterogeneity in AD drug repurposing. Despite these encouraging results, several limitations should be acknowledged. First, we used only the 5xFAD mouse model, which primarily represents a familial AD and may not fully recapitulate sporadic or age-related AD pathology. Second, although ADAM10 upregulation was observed, downstream markers such as sAPPα or synaptic proteins were not comprehensively evaluated. Third, dose–response relationships and potential off-target effects were not assessed. In addition, direct measurements of Aβ production in cell culture were not performed, which limiting conclusions regarding causal linkage between signaling modulation and amyloid generation. Finally, only female animals were evaluated. Given the known sex-dependent differences in 5XFAD pathology and recognized sex differences in human AD, future studies including male cohorts will be necessary to determine generalizability.

In summary, our findings provide evidence supporting the involvement of GSK3β–CREB–ADAM10 signaling in fluoxetine-associated molecular changes in 5xFAD mice. These preclinical results suggest that fluoxetine may influence amyloid-related and inflammatory processes; however, further mechanistic and translational studies will be necessary to determine its potential clinical applicability in Alzheimer’s disease.

## 4. Materials and Methods

### 4.1. Mouse Experiment and Drug Administration

Heterozygous 5xFAD transgenic (Tg) and wild type (WT) mice were obtained by cross breeding male 5xFAD Tg and SJL/B6 F1 female mice from the Jackson Laboratory (Bar Harbor, ME, USA). At 5 months of age, female 5xFAD mice were allocated to either the vehicle control group (5xFAD + Veh, *n* = 6) or the fluoxetine-treated group (5xFAD + Fluo, *n* = 6). Age-matched female WT mice were also assigned to vehicle- (*n* = 6) or fluoxetine-administration (*n* = 5). Given that beta-amyloid accumulation and neuroinflammation tend to be more severe in female 5xFAD mice [35], only female 5xFAD Tg and WT mice were used.

**Figure 7:**
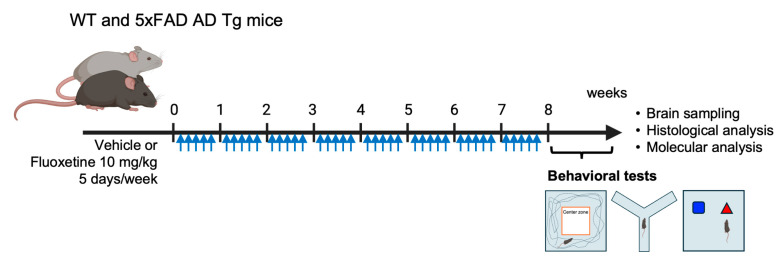
Experimental design. Five-month-old wild-type (WT) and 5xFAD Transgenic mice were administered intraperitoneal injections of 10 mg/kg fluoxetine (Fluo) or vehicle (Veh), five times a week for 8 weeks. At 7 months of age, the animals underwent a series of behavioral tests, followed by sacrifice and brain collection for molecular and histopathologic analyses. Created in BioRender. Son, Y. (2026) https://BioRender.com/s91n646 (accessed on 31 January 2026).

Fluoxetine (Tokyo Chemical Industry, Tokyo, Japan) was dissolved in 5% dimethyl sulfoxide in phosphate-buffered saline (PBS). At 5 months of age, fluoxetine (10 mg/kg) was intraperitoneally administered to the mice for 8 weeks (Figure 7). All mice were maintained in a specific pathogen-free environment at 22 ± 2 °C and 60% ± 5% relative humidity, under a 12:12 h light/dark cycle with unrestricted access to normal diet and autoclaved water. All animal procedures were approved by the Institutional Animal Care and Use Committee of the Korea Institute of Radiological and Medical Sciences (IACUC approval number: KIRAMS2020-0077).

### 4.2. Behavior Test

#### 4.2.1. Open-Field Test

The open-field test was performed to evaluate both spontaneous locomotor activity and anxiety-associated behavior. Mice were placed individually in an open-field chamber consisting of a 45 cm (W) × 45 cm (L) × 30 cm (H) acrylic box with opaque walls, and their movements were recorded over a 10 min period. Key parameters included total distance traveled and the duration spent in the central area. Behavioral tracking was conducted using an automated video analysis system (Viewer3, Biobserve, Bonn, Germany). The blue squares and red triangles represent familiar and novel objects, respectively, as described previously. Blue arrows indicate the schedule of drug administration (5days/week).

#### 4.2.2. Y-Maze Test

The Y-maze test was used to evaluate the spatial working memory by measuring spontaneous alternation behavior. An alternation was defined as sequential entries into all three arms without repetition, and the alternation percentage was calculated based on the total possible alternations during an 8 min session. Alternation, a measure of memory, was calculated as the percentage of actual alternations out of all possible alternations (see Equation (1))

(1) $$\%\;alternation=\frac{number\;of\;alternation}{Total\;arm\;enties-2}\;\times \;100$$

#### 4.2.3. Novel Object Recognition Test

Recognition memory was assessed using the novel object recognition test. Mice were habituated to the testing chamber over three consecutive days. During the training phase, each mouse was exposed to two identical objects for 10 min. After 24 h, one of the familiar objects was replaced with a novel object for the test session. Exploration was defined as the animal orienting its nose toward the object in close proximity or making physical contact. To avoid olfactory cue accumulation, 70% (*v*/*v*) ethanol was used to clean the chamber and all items in between the trials. The discrimination ratio (DR), reflecting the preference for the novel object, was calculated as the difference in exploration time between the novel (T (new)) and familiar (T (new)) objects relative to the total exploration time (see Equation (2)).

(2) $$Discrimination\;ratio=\frac{\begin{array}{c} T\;(\mathrm{new})-T\;(\mathrm{old}) \end{array}}{T\;(total)}$$

### 4.3. Immunohistochemistry

Paraffin embedded brain sections were rehydrated using a series of graded ethanol solutions after deparaffinization with xylene. For antigen retrieval, the sections were heated in boiling water in a citrate buffer (pH 6.0) for 30 min. The tissue slides were blocked for 30 min with 1.5% normal goat serum (Vector ABC Elite Kit, Vector Laboratories Inc., Burlingame, CA, USA) to avoid nonspecific staining and then incubated overnight with anti-6E10 (Biolegend, San Diego, CA, USA; Sig-39320, 1:1000) at 4 °C. The slides were washed with 0.1% TritonX-100 in PBS (PBS-T) and treated with biotinylated secondary antibodies (Vector Laboratories Inc.) for 30 min at room temperature. The slides were then incubated for 30 min at room temperature with an avidin–biotin–peroxidase complex (Vector Laboratories) and subsequently with diaminobenzidine (DAB, Vector Laboratories) for immunohistochemistry. Whole brain images were captured at 20× magnification using a digital slide scanner (Aperio VERSA; Leica Biosystems, Wetzlar, Germany), and the percentage of positive area or positive cells was quantified using the Aperio image analysis software v12.4.6.

### 4.4. Immunofluorescence Staining

Following deparaffinization and rehydration, antigen retrieval was performed using citrate buffer (pH 6.0). Tissue sections were then incubated in a blocking solution containing normal goat serum, followed by overnight incubation at 4 °C with primary antibodies: anti-6E10 (Biolegend; Sig-39320, 1:1000) and anti-Iba-1 (Abcam, Cambridge, UK; ab-15690, 1:1000) overnight. On the next day, sections were incubated for 30 min at room temperature with fluorescence-conjugated secondary antibodies: Alexa Fluor 488 donkey anti-rabbit (Invitrogen, Carlsbad, CA, USA; A-21202, 1:200) and Alexa Fluor 546 donkey anti-mouse (Invitrogen; A-10040, 1:200). After washing, nuclei were counterstained with DAPI, and mounted with an anti-fade mounting medium. Fluorescence images were acquired using a BX-53 microscope (Olympus, Tokyo, Japan) equipped with a DP73 CCD camera.

### 4.5. Cell Culture

SH-SY5Y cells were obtained from Korean Cell Line Bank (Seoul, Republic of Korea) and cultured in minimal essential medium (Welgene, Gyeongsan-si, Gyeongsangbuk-do, Republic of Korea) with 10% fetal bovine serum (Corning, NY, USA) and 1% antibiotic–antimycotic at 37 °C in a humidified 5% CO_2_ incubator. The medium was changed every three days.

### 4.6. Drug and Inhibitor Treatment

Cells were seeded at a density of 6 × 10^6^ cells in a 100 mm cell culture dish. After 24 h, 10 µM fluoxetine was added to the culture dishes for 12 h. 1-Naphthyl PP1 (1-NA-PP1, HY-13941), a CAMKII inhibitor, and GSK3 inhibitor IX (HY-10580), a GSK3β inhibitor, were purchased from MedChem Express (Princeton, NJ, USA). The cells were treated with the inhibitors for 1 h to inhibit the respective signaling pathway.

### 4.7. RNA Extraction and RT-qPCR

Total RNA was isolated using the TRIzol reagent (#79306, Qiagen, Valencia, CA, USA). RNA quality was assessed using an Agilent 2100 Bioanalyzer with an RNA 6000 Nano Chip (Agilent Technologies, Amstelveen, The Netherlands), and RNA quantification was performed using an ND-2000 Spectrophotometer (Thermo Inc., Waltham, MA, USA). The sequences of the primers used were as follows: GAPDH–forward: 5′-CAAGAAGGTGGTGAAGCAGG-3′, reverse: 5′-AGGTGGAAGAGTGGGAGTTG-3′, IL-6–forward: 5′-CCTTCCCTACTTCACAAGTC-3′, reverse: 5′-ACCTTTTGACAGTGATGAGAA-3′ and TNF-α–forward: 5′-TTGTACCTTGTCTACTCCCA-3′, reverse: 5′-AGACTCCTCCCAGGTATATG-3′.

### 4.8. Protein Extraction

A Pro-prep solution (iNtRon, Anseong-si, Gyeonggi-do, Republic of Korea; #17081) was used to homogenize the minced hippocampus. Bradford reagent (Bio-Rad, Hercules, CA, USA) was used to measure the protein concentration in the resultant homogenate. To extract proteins from SH-SY5Y cells, cell culture dishes were washed twice with DPBS (Welgene) and RIPA lysis buffer (MBiotech, Hanam-si, Gyeonggi-do, Republic of Korea) was added for cell lysis. The buffer was collected in 1.5 mL microcentrifuge tube and incubated on ice for 5 min, followed by centrifugation at 13,000 rpm, 4 °C for 5 min. The protein content was measured using the Bradford assay (Bio-Rad, Hercules, CA, USA), following the manufacturer’s protocol. The samples were denatured by boiling for 5 min.

### 4.9. Western Blotting

Sodium dodecyl sulfate-polyacrylamide gel electrophoresis was used to separate the proteins in each sample, with gel percentages varying from 8% to 15%. After separation, the proteins were transferred onto nitrocellulose membranes. The membranes were incubated for 30 min in a 5% bovine serum albumin solution to prevent nonspecific binding. Thereafter, the membranes were incubated overnight with primary antibodies against different target proteins at 4 °C: anti-6E10 (Biolegend; Sig-39320, 1:1000), anti-ADAM10 (Abcam, Cambridge, UK; ab1997, 1:1000), anti-ADAM17 (Genetex, San Antonio, TX, USA; GTX31632, 1:1000), anti-BACE1 (Santa Cruz Biotechnology, Dallas, TX, USA; sc-33711, 1:1000), anti-Iba1 (Abcam, ab15690, 1:1000), anti-CREB (Cell Signaling Technology, Danvers, MA, USA; #9197, 1:1000), anti-p-CREB (Cell Signaling Technology; #9198, 1:1000), GSK-3β (Cell Signaling Technology; #9315, 1:1000), anti-p-GSK-3β (Cell Signaling Technology; #9322, 1:1000), anti-CAMKII (Genetex; GTX133071, 1:1000), anti-p-CAMKII (Genetex; GTX22724, phospho Thr286, 1:1000), and anti-β-actin (Cell Signaling; #3700, 1:3000). The membranes were carefully washed with PBS-T and then incubated for 1 h with diluted corresponding secondary antibodies (1:3000). After an additional washing step with PBS-T, an enhanced chemiluminescence kit (Perkin Elmer, Waltham, MA, USA; NEL105001EA) was used to detect immunoreactivity

### 4.10. Statistical Analysis

All data are presented as mean ± standard error of the mean (SEM). Statistical analyses were performed using GraphPad Prism (version 10.6, GraphPad Software, San Diego, CA, USA). For comparisons among more than two groups, one-way ANOVA followed by Tukey’s post hoc test was used to determine statistical significance. When comparisons were restricted to two experimental groups, an unpaired two-tailed Student’s *t*-test was applied. For analyses involving 6E10 immunoreactivity (Figure 2B) and corresponding Western blot quantification (Figure 2D), wild-type (WT) groups exhibited negligible background-level signal. Therefore, statistical comparisons were performed only between AD Tg + vehicle and AD Tg + fluoxetine groups using an unpaired two-tailed Student’s *t*-test. The number of biological replicates (n) for each experiment is specified in the corresponding figure legends. Each biological replicate represents an individual animal (in vivo experiments) or an independent cell culture experiment (in vitro experiments). A *p*-value < 0.05 was considered statistically significant.

## Data Availability

The original contributions presented in this study are included in the article. Further inquiries can be directed to the corresponding authors.
